# Aerobic performance of two tropical cephalopod species unaltered by prolonged exposure to projected future carbon dioxide levels

**DOI:** 10.1093/conphys/coz024

**Published:** 2019-06-07

**Authors:** Blake L Spady, Tiffany J Nay, Jodie L Rummer, Philip L Munday, Sue-Ann Watson

**Affiliations:** 1 Australian Research Council Centre of Excellence for Coral Reef Studies, James Cook University, Townsville, QLD, Australia; 2 College of Science and Engineering, James Cook University, Townsville, QLD, Australia; 3 Biodiversity and Geosciences Program, Museum of Tropical Queensland, Queensland Museum, Townsville, Queensland, Australia

**Keywords:** Aerobic scope, cephalopod, CO_2_ respirometry, ocean acidification, oxygen uptake

## Abstract

Squid and many other cephalopods live continuously on the threshold of their environmental oxygen limitations. If the abilities of squid to effectively take up oxygen are negatively affected by projected future carbon dioxide (CO_2_) levels in ways similar to those demonstrated in some fish and invertebrates, it could affect the success of squid in future oceans. While there is evidence that acute exposure to elevated CO_2_ has adverse effects on cephalopod respiratory performance, no studies have investigated this in an adult cephalopod after relatively prolonged exposure to elevated CO_2_ or determined any effects on aerobic scope. Here, we tested the effects of prolonged exposure (≥20% of lifespan) to elevated CO_2_ levels (~1000 μatm) on the routine and maximal oxygen uptake rates, aerobic scope and recovery time of two tropical cephalopod species, the two-toned pygmy squid, *Idiosepius pygmaeus* and the bigfin reef squid, *Sepioteuthis lessoniana*. Neither species exhibited evidence of altered aerobic performance after exposure to elevated CO_2_ when compared to individuals held at control conditions. The recovery time of *I. pygmaeus* under both control and elevated CO_2_ conditions was less than 1 hour, whereas *S. lessoniana* required approximately 8 hours to recover fully following maximal aerobic performance. This difference in recovery time may be due to the more sedentary behaviours of *I. pygmaeus*. The ability of these two cephalopod species to cope with prolonged exposure to elevated CO_2_ without detriment to their aerobic performance suggests some resilience to an increasingly high CO_2_ world.

## Teaser

Long-term exposure to projected future CO_2_ levels has no effect on the aerobic performance of two tropical cephalopod species. The resting oxygen uptake rates, maximal oxygen uptake rates, aerobic scope, and recovery time of both bigfin reef squid and two-toned pygmy squid were unaltered by elevated CO_2_.

## Introduction

Atmospheric carbon dioxide (CO_2_) concentrations have increased from 280 ppm before the industrial revolution to over 400 ppm in the current day (Dlugokencky and Tans, 2018), a level that is higher than any time in the past 800 000 years (Lüthi *et al.*, 2008). On the current emissions trajectory, it is projected that atmospheric CO_2_ will exceed 900 ppm by the end of this century (Collins *et al.*, 2013), increasing at a rate at least an order of magnitude faster than at any time in the past million years (Doney and Schimel, 2007). The partial pressure of CO_2_ (*p*CO_2_) in the surface oceans is in approximate gas equilibrium with the atmosphere, meaning that CO_2_ concentrations in the oceans are increasing at approximately the same rate as the atmosphere (Doney, 2010). Furthermore, the oceans experience seasonal fluctuations in *p*CO_2_, and these fluctuations are projected to become amplified in the future due to the increased Revelle (buffer) factor of acidified seawater (McNeil and Sasse, 2016). The increase in average *p*CO_2_ along with the amplification of seasonal cycles of *p*CO_2_ indicates that marine organisms could experience CO_2_ levels >1000 μatm before the end of the century (McNeil and Sasse, 2016). This rapid increase of CO_2_ levels in the surface oceans could have a range of adverse effects on many marine species (Hoegh-Guldberg *et al.*, 2007; Fabry *et al.*, 2008; Doney *et al.*, 2009; Kroeker *et al.*, 2013; Clements and Hunt, 2015; Cattano *et al.*, 2018).

The capacity to deliver sufficient oxygen to the tissues, in order to meet increasing demand, has been hypothesized to constrain the performance of marine species under climate change and ocean acidification (Fry and Hart, 1948; Pörtner and Farrell, 2008). Squid, the most active order of cephalopods, have high mass-specific oxygen consumption rates and blood with low oxygen-carrying capacity when compared to fishes (O’Dor and Webber, 1986; Shadwick *et al.*, 1990). One of their most advantageous evolutionary adaptations, jet propulsion, allows them to rapidly escape predators and compete for food with carnivorous fishes (Hanlon and Messenger, 1996). However, this mode of locomotion is inherently inefficient, requiring a significant output of energy from their mantle muscles, which further increases their already high demand for oxygen (O’Dor, 1988a, 1988b). To satisfy such a high demand of oxygen, both at rest and during sustained swimming, squid must pump large amounts of blood and extract most (or all) of the oxygen from it during one cycle through the body, leaving little to no venous oxygen reserve (O’Dor and Webber, 1986; Wells *et al.*, 1988; Rosa and Seibel, 2008). The combination of these factors contributes to many squid species living chronically at the threshold of their oxygen limitation (Pörtner, 2002). Furthermore, the oxygen-carrying capacity of cephalopod hemocyanin, which delivers the oxygen to tissues and cells, has been hypothesized to be highly sensitive to changes in pH (Bridges, 1995). These unique physiological traits may mean the respiratory capacity of squid is affected by rising CO_2_ levels in the ocean. However, models by Birk *et al*. (2018) suggest that climate change relevant CO_2_ levels may not alter squid blood oxygen binding to a degree that will affect aerobic performance. Whether the oxygen uptake capabilities of squid will be susceptible to elevated CO_2_ levels may depend on the level of sensitivity of their hemocyanin to changes in pH (e.g. Seibel, 2016; Birk *et al*., 2018).

The physiological performance of aquatic animals has often been linked to their aerobic scope, the difference between the routine oxygen uptake (*Ṁ*O_2Routine_) and maximal oxygen uptake (*Ṁ*O_2Max_) rates. Aerobic scope indicates the amount of oxygen available, beyond basal metabolic costs, for critical aerobic activities (Eliason *et al.*, 2008; Pörtner and Farrell, 2008; Pörtner and Peck, 2010). The type of effect and magnitude of changes in aerobic scope resulting from elevated CO_2_ appear to be species specific. In fishes, for example, the effects of elevated CO_2_ on aerobic scope are highly variable, with different studies reporting a decrease (Munday *et al.*, 2009), increase (Couturier *et al.*, 2013; Rummer *et al.*, 2013) or no effect on aerobic scope (Melzner *et al.*, 2009a). Moreover, recent meta-analyses find no consistent effect of elevated CO_2_ on aerobic scope in marine fishes when all studies conducted to date are considered (Lefevre, 2016; Cattano *et al.*, 2018; Hannan and Rummer, 2018). Although less studied, the reported effects of elevated CO_2_ on aerobic scope in molluscs are also variable. Elevated CO_2_ causes a reduced aerobic scope in the scallop, *Pecten maximus* (Schalkhausser *et al.*, 2013), but no effect on the aerobic scope of the gastropod, *Gibberulus gibberulus gibbosus* (Watson *et al*., 2014; Lefevre *et al.*, 2015). The effects of elevated CO_2_ on resting or routine oxygen consumption also varies among mollusc species. Elevated CO_2_ causes a depression in oxygen uptake rates in six bivalve species (Fernández-Reiriz *et al.*, 2011; Wenguang and Maoxian, 2012; Navarro *et al.*, 2013) and one gastropod species (Melatunan *et al.*, 2011). However, other bivalve species respond to elevated CO_2_ with an increase in oxygen uptake rates (Beniash *et al.*, 2010; Cummings *et al.*, 2011). Given the variety of effects on respiratory performance observed in a range of mollusc taxa, most of those being sedentary species, it is difficult to predict how the more active cephalopod species will respond to elevated CO_2_.

In cephalopods, the effects of elevated CO_2_ on respiratory performance appear to be both species as well as life stage specific. Very high CO_2_ (~4000 μatm) had no effect on oxygen consumption in juvenile common cuttlefish, *Sepia officinalis* (Gutowska *et al.*, 2008). In the same species, similarly high CO_2_ exposure (~3600 μatm) reduced oxygen uptake by ~ 20% during the late-stage embryo incubation period, although there was no effect at CO_2_ levels more closely resembling those that could occur in the ocean in the next 100 years (~1400 μatm) (Sigwart *et al.*, 2016). Elevated CO_2_ (~1650 μatm) reduced oxygen uptake rates in late-stage embryos and newly hatched paralarvae of the squid *Loligo vulgaris* (Rosa *et al*., 2014). By contrast, *Ṁ*O_2Routine_ of adult bigfin reef squid (*Sepioteuthis lessoniana*) was not altered by moderately high CO_2_ levels (1586 μatm) (Hu *et al.*, 2014), yet oxygen uptake rates were depressed by 40% when they were exposed to very high CO_2_ levels (4134 μatm) for 7 days (Hu *et al.*, 2014). Juvenile jumbo squid, *Dosidicus gigas*, showed suppressed *Ṁ*O_2_ at elevated CO_2_ levels (estimated at ~ 1000 μatm) (Rosa and Seibel, 2008); however, with a longer acclimation to CO_2_ and the use of intermittent-flow respirometry rather than flow-through respirometry, there was no effect of elevated CO_2_ (1410 μatm) on the species (Birk *et al*., 2018). These results indicate a range of possible effects of elevated CO_2_ on the respiratory capacity of cephalopods that may be dependent on species and life stage. However, it also appears that prolonged exposure to elevated CO_2_ and using appropriate respirometry techniques may be important in establishing the physiological effects of rising ocean CO_2_ levels on cephalopods.

In this study, we tested the effects of projected future CO_2_ levels on the respiratory performance of two tropical cephalopod species. The two-toned pygmy squid (*Idiosepius pygmaeus*) has an average body length of < 20 mm and inhabits shallow coastal waters from northern Australia to the South China Sea (Moynihan, 1983; Semmens *et al.*, 1995). This species has a unique adhesive gland on the mantle, allowing it to attach to seagrass or flotsam where it can rest for extended periods (von Byern and Klepal, 2006). These seagrass habitats in which *I. pygmaeus* can be found have the potential for diel fluctuations in CO_2_ concentrations between ~ 80 and 700 μatm (Chou *et al*., 2018), indicating that the species may be able to tolerate periods of elevated CO_2_ conditions. The second species, the bigfin reef squid, is a larger and much more active species, found in tropical waters of up to 100 m, and individuals are often found feeding in coral reef habitats at night (Norman, 2003). These two species, while both commonly called `squid’, are in fact from two separate taxonomic orders. The bigfin reef squid is a true squid of the order Teuthida, whereas the pygmy squid is in the order Idiosepiida, which is more closely related to cuttlefish (Sepiida). The two species were subjected to a current-day control (396–440 μatm) or a projected future CO_2_ level (997–1039 μatm) for 18 (pygmy squid) or 75 (bigfin reef squid) days. Pygmy squid and bigfin reef squid live for ~90 and 210 days, respectively; therefore, the treatment period was ~20 and 36% of the total lifespan of each species. We hypothesized that elevated CO_2_ would reduce *Ṁ*O_2Max_ and aerobic scope in both cephalopod species, possibly due to the pH sensitivity of blood oxygen binding in some active squids (Bridges, 1995). To test this, we measured *Ṁ*O_2Max_ and *Ṁ*O_2Routine_ via intermittent-flow respirometry and then calculated aerobic scope (*Ṁ*O_2Max_—*Ṁ*O_2Routine_) in both the pygmy squid and bigfin reef squid after prolonged exposure to global change relevant levels of elevated CO_2_.

## Materials and methods

### CO_2_ treatment systems

Experiments were conducted using 8000 l recirculating seawater systems at James Cook University’s research aquarium in Townsville, Australia. CO_2_ levels were set at (i) a current-day control (*I. pygmaeus*, 396 μatm; *S. lessoniana*, 440 μatm) and (ii) an upper end-of-century projection following RCP8.5 (elevated CO_2_: *I. pygmaeus*, 1039 μatm; *S. lessoniana*, 997 μatm) (Collins *et al.*, 2013). A pH control system (AT Control; Aqua Medic, Germany) dosed CO_2_ into 3000 l sumps to achieve the desired pH level for each CO_2_ treatment. pH on the National Bureau of Standards (NBS) scale (pH_NBS_) was measured daily (Seven2Go Pro; Mettler Toledo, Switzerland), and dosing set points were adjusted as necessary to maintain the target *p*CO_2_ in each treatment. Equilibrated seawater from each system was delivered at a rate of 1.5 l min^−1^ to tanks containing squid. Temperature was measured daily in each tank (Comark C26; Norfolk, UK).

Water samples were taken weekly to determine pH on the total scale (pH_T_) by spectrophotometry (UVmini-1240; Shimadzu, Suzhou Instruments Co. Ltd, Kyoto, Japan) using m-cresol purple as an indicator dye (Dickson and Millero, 1987; Dickson *et al.*, 2007). Comparison of pH_NBS_ and pH_T_ in the weekly sample were used to estimate daily pH_T_ values. Total alkalinity was estimated weekly by Gran Titration (888 Titrando; Metrohm AG, Switzerland) ensuring titration calibrations remained within 1% of certified reference material from Dr A.G. Dickson (Scripps Institution of Oceanography, batch #135). Salinity was measured weekly using a conductivity sensor (HQ15d; Hach, Loveland, CO, USA). Carbonate chemistry parameters (Table 1) were calculated in CO2SYS (Pierrot *et al.*, 2006) using the constants K1 and K2 from Mehrbach *et al.* (1973) refit by Dickson and Millero (1987) and Dickson *et al.* (2007) for KHSO_4_.

**Table 1:** Mean seawater data (± SD) for each species; total alkalinity and salinity values are from weekly measurements

Species	CO_2_ treatment	Temperature (°C)	Salinity	pH_(T)_	Total alkalinity (μmol/kg SW)	*p*CO_2_ (μatm)
*I. pygmaeus*	Control	28.1 (±0.3)	36.7 (±1.1)	8.05 (±0.07)	2373 (±55)	396 (±76)
	Elevated	28.1 (±0.4)	36.4 (±0.9)	7.67 (±0.05)	2228 (±63)	1039 (±118)
*S. lessoniana*	Control	28.2 (±0.4)	36.3 (±0.4)	7.99 (±0.05)	2229 (±67)	440(±60)
	Elevated	28.5 (±0.4)	36.0 (±0.7)	7.69 (±0.04)	2212 (±130)	997 (±110)

### Experimental animals

Two-toned pygmy squid (wet mass, 0.25 ± 0.09 g; mantle length, 11.2 ± 1.7 mm; means ± SD) were collected by dip net (500 μm mesh) in March 2017 from Cleveland Bay in Townsville, Queensland, Australia (19°24′S, 146°82′E) and immediately transported to James Cook University, Townsville. Squid were maintained in round tanks (47Ø × 51H cm) filled with seawater to 67 l, at a maximum of five individuals per tank. Animals were provided with PVC pipe structures in the tanks as shelter. Animals were maintained at control conditions for 24 hours in holding tanks before being transferred to separate tanks of the same size that received a continuous flow of either control or elevated CO_2_ treatment water. Glass shrimp (*Acetes sibogae australis*) were provided *ad libitum* to squid every morning. Squid were observed regularly feeding throughout the day, but food was withheld for 24 hours prior to experimentation to ensure a post-absorptive state that would maximize energy available for performance (Niimi and Beamish, 1974). Pygmy squid remained in either control (*n* = 8) or elevated CO_2_ (*n* = 10) treatment for 18 days before respirometry trials. A treatment duration of 18 days was chosen for pygmy squid because their brief lifespans mean that mature animals could only be expected to live a maximum of 30 (females) to 45 (males) days post-capture (Jackson, 1988). This treatment duration represents ~20% of the total pygmy squid lifespan.

Bigfin reef squid (wet mass, 181.2 ± 39.9 g; mantle length, 148.7 ± 13.7 mm; means ± SD) were collected in June 2016 from the Townsville breakwater, Queensland, Australia. Animals were captured at night with a 2.5 cm mesh, round frame dip net and transported immediately to James Cook University, Townsville. Squid were kept individually in round tanks (47Ø × 51H cm) filled to 67 l. Individuals were kept at control conditions for 21 days before they were transferred to separate tanks of the same size that received a continuous flow of either control or elevated CO_2_ treatment water. Bigfin reef squid were fed a variety of live food, including locally caught estuary glassfish (*Ambassis marianus*), juvenile flathead grey mullet (*Mugil cephalus*), as well as spiny chromis damselfish (*Acanthochromis polyacanthus*), twice daily. Feeder fish were maintained under control CO_2_ conditions prior to being offered to the squid. Bigfin reef squid remained in either control (*n* = 9) or elevated CO_2_ (*n* = 7) treatment for 75 days before their respirometry trials. In comparison to the pygmy squid, we chose a longer treatment duration for the bigfin reef squid because of their greater longevity. This treatment duration represents ~35% of their average 208-day lifespan in the wild (Walsh *et al.*, 2002).

### Maximal and routine oxygen uptake measurements

Intermittent-flow respirometry (Clark *et al.*, 2013) was used to determine both routine oxygen uptake (*Ṁ*O_2Routine_) and maximal oxygen uptake (*Ṁ*O_2Max_) for both cephalopod species. The *Ṁ*O_2Max_ was established by using a standard exercise challenge immediately before placing the animal in the respirometry chamber. The use of a swim chamber would not have proved effective in determining *Ṁ*O_2Max_ for either species. Pygmy squid tend to attach to the sides of the chamber with their unique adhesive mantle gland rather than swimming against the flow within a swimming chamber. Bigfin reef squid can unpredictably switch locomotory modes from fin undulations to jet propulsion, which can result in self-injury if they propel themselves against the chamber while swimming actively. Therefore, to achieve *Ṁ*O_2Max_ for pygmy squid, animals were chased with a dip net in a small circular tank for 3 minutes, followed by a 15 second air exposure period (Roche *et al.*, 2013; Rummer *et al.*, 2016) immediately prior to introduction to the respirometry chamber. Pygmy squid (all individuals) were unable to continue jet escaping the dip net before the end of the 3-minute period due to exhaustion. The number of jets and ink discharges from each animal during the chase period were recorded. For the bigfin reef squid, however, due to potential injury reasons mentioned above, a different method was used to elicit *Ṁ*O_2Max_. Instead, bigfin reef squid were held in a large dip net and periodically lifted in and out of the water (10 seconds in/5 seconds out) for 3 minutes. On re-immersions into the water, squid would attempt to jet escape 0–4 times within the safety of the net. All squid ceased jetting before the end of the 3 minutes of periodic emersions. Bigfin reef squid were then subjected to a further 30-second air exposure period before being placed into respirometry chambers. The number of jets and ink discharges were also recorded.

The measurement period for intermittent-flow respirometry (time during which the flush pump was off) was determined as the minimum time required to ensure a steady slope representing the decline in O_2_ concentration of the chamber down to 75–80% air saturation over time (O_2_ uptake rate of the squid). The flush period (time during which the flush pump was on) was sufficient time for the O_2_ concentration of the chambers to be sufficiently replenished back to ~ 100%. Pygmy squid were tested in 20 ml chambers submerged in an aquarium with continuous delivery of water from their CO_2_ treatment system, with both flushing and recirculation pumps submerged and providing flow at 21.6 l h^−1^. Preliminary experiments determined that *Ṁ*O_2Routine_ of pygmy squid was reached in under 2 hours, so total trial time was set at 4 hours (Fig. S1). The measurement period was set at 150 seconds followed by a 60-second flushing period. Bigfin reef squid were tested in 6840 ml chambers also submerged in continuously replenished water from the CO_2_ treatment system of the squid being tested. The flush and recirculation pumps for these chambers delivered water at 400 l h^−1^. Measurement periods for bigfin reef squid lasted 85 seconds, followed by a 300-second flush period; this cycle continued for a total 22-hour trial period. The squid to chamber volume ratio was between 1:20 and 1:50 for all animals and the O_2_ concentration did not fall below 80% air saturation during measurement periods in any of the chambers for either species (Svendsen *et al.*, 2016).

Temperature-compensated O_2_ concentration was continuously recorded (0.5 Hz) using oxygen-sensitive REDFLASH dye on contactless spots (2 mm) adhered to the inside of a cut glass pipette tube set within the recirculation pump loop and linked to a Firesting Optical Oxygen Meter (Pyro Science e.K., Aachen, Germany) via fibre-optic cables. Data were analysed in LabChart version 8.1.3 (ADInstruments, Colorado Springs, CO, USA), and *Ṁ*O_2_ (in milligrammes O_2_ per kilogramme of animal per hour) was calculated as the slope of the linear regression of oxygen concentration decline over time during the measurement period using the following equation:

\begin{equation*} \dot{M}O_{2} = SV_{\textrm{resp}}M^{-1}, \end{equation*}

where *S* is the slope (in milligrammes of O_2_ per litre per second), *V*_resp_ is the volume of the respirometer minus the volume of the squid (in litres) and *M* is the mass of the squid (in kilogrammes). The volume of respirometry chambers included the volume of the chamber as well as that of the recirculation tubing and pump. The value of *Ṁ*O_2Routine_ was calculated by taking the average of the lowest 10% of *Ṁ*O_2_, minus the background O_2_ uptake, which was measured before and after each trial (assumed linear) (Rummer *et al.*, 2016). The *Ṁ*O_2Max_ was calculated by isolating the first five slopes into segments (each segment being 20% of the total measurement period) and selecting the highest rate of change found therein and ensuring that the R^2^ of slopes was above 0.95. Aerobic scope for each individual was calculated by subtracting the *Ṁ*O_2Routine_ value from the *Ṁ*O_2Max_ value. Recovery time was defined as the amount of time, from introduction to the chamber, for *Ṁ*O_2_ to first reach an equal or lesser value of the *Ṁ*O_2Routine_ value for that individual. To restrict background respiration to <5% of a squid’s *Ṁ*O_2Routine_, chambers and pumps were rinsed with fresh water and 10% bleach solution after each trial and left to dry for 12 hours before being used again.

**Figure 1:**
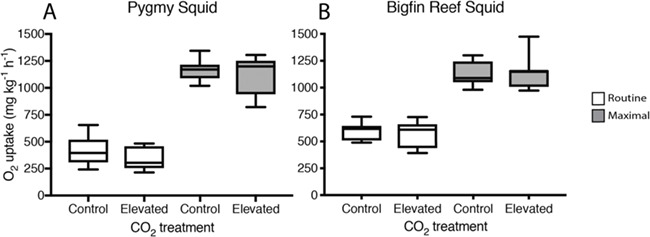
Routine (white boxes) and maximal oxygen uptake (grey boxes) of pygmy squid (A) and bigfin reef squid (B) from current day control and elevated CO_2_ treatments; boxplots show the median and interquartile range and the minimum and maximum range of the data.

**Figure 2:**
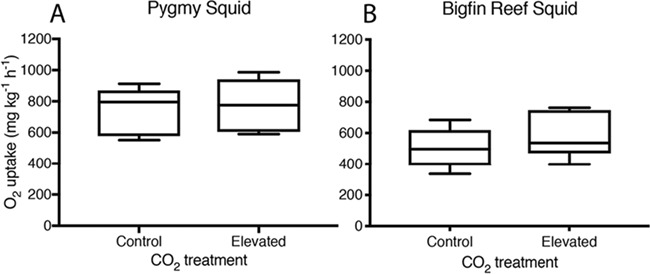
Absolute aerobic scope of pygmy squid (A) and bigfin reef squid (B) from current day control and elevated CO_2_ treatments; boxplots show the median and interquartile range and the minimum and maximum range of the data.

**Figure 3:**
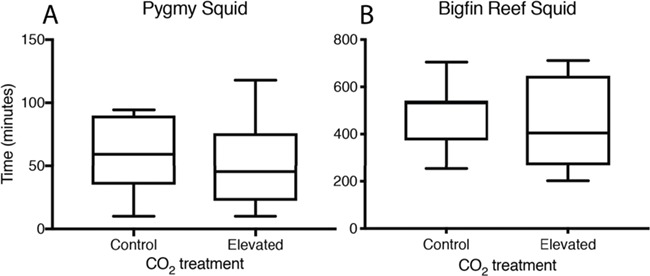
Recovery time that pygmy squid (A) and bigfin reef squid (B) required to reach a stable routine oxygen uptake rate following exhaustive exercise at current-day control and elevated CO_2_ treatment levels; boxplots show the median and interquartile range and the minimum and maximum range of the data (Note: the *y*-axis for pygmy squid ranges from 0–150 minutes while the *y*-axis for bigfin reef squid ranges from 0–800 minutes).

### Statistical analyses

Generalized linear mixed models with Gaussian distributions were used to compare response variables (*Ṁ*O_2Routine_, *Ṁ*O_2Max_, aerobic scope and recovery time) for pygmy squid between CO_2_ treatments, with the number of jets and number of inks included as fixed factors and holding tank included as a random effect. Linear models with log-transformed data were used to compare the same response variables of bigfin reef squid between CO_2_ treatments, with number of jets and inks included as fixed factors. A tank effect was not applicable for bigfin reef squid as they were always housed individually. A linear model with square root transformed data tested for differences in recovery times between the two squid species.

Statistical analyses were performed with R statistical software (R Development Core Team, 2018). Residual analysis indicated that data met the assumptions of normality and homogeneity of variance. Power analyses were performed to determine the probability of finding a significant difference in aerobic scope given the sample size and effect size.

## Results

### Pygmy squid

Elevated CO_2_ did not affect any of the traits measured for pygmy squid. Pygmy squid had a mean average *Ṁ*O_2Max_ of 1164 ± 57 mg kg^−1^ h^−1^ (mean ± SE) under control and 1117 ± 35 mg kg^−1^ h^−1^ under elevated CO_2_ (*x*^2^ = 0.112, df = 14, *P* = 0.738) conditions. The *Ṁ*O_2Routine_ was 341 ± 33 mg kg^−1^ h^−1^ under control and 418 ± 47 mg kg^−1^ h^−1^ under elevated CO_2_ and was not significantly different between treatments (*x*^2^ = 2.667, df = 14, *P* = 0.102) (Fig. 1A). The aerobic scope of pygmy squid was similar between CO_2_ treatments, with an average of 746 ± 52 mg kg^−1^ h^−1^ at control compared to 776 ± 49 mg kg^−1^ h^−1^ under elevated CO_2_ (*x*^2^ = 0.929, df = 14, *P* = 0.335) (Fig. 2A). Recovery time of pygmy squid under both control and elevated CO_2_ conditions lasted for just under 1 hour, averaging 55 minutes and 12 seconds (±10 minutes and 42 seconds) under control CO_2_ and 53 minutes and 9 seconds (±11 minutes and 48 seconds) under elevated CO_2_ conditions (*x*^2^ = 0.006, df = 14, *P* = 0.936) (Fig. 3A). A power analysis revealed a power of 0.067 on the comparisons of aerobic scope in pygmy squid between CO_2_ treatments.

### Bigfin reef squid

As observed in pygmy squid, elevated CO_2_ did not affect any of the traits measured for bigfin reef squid. The average *Ṁ*O_2Max_ of bigfin reef squid under both control and elevated CO_2_ treatment levels were very similar at 1133 ± 36 mg kg^−1^ h^−1^ and 1139 ± 63 mg kg^−1^ h^−1^, respectively (*x*^2^ = 0.011, df = 14, *P* = 0.918). Average *Ṁ*O_2Routine_ measurements between treatments were also similar at 596 ± 27 mg kg^−1^ h^−1^ in the control and 565 ± 46 mg kg^−1^ h^−1^ in elevated CO_2_ (*x*^2^ = 0.588, df = 14, *P* = 0.443) (Fig. 1B). The aerobic scope of bigfin reef squid averaged 509 ± 41 mg kg^−1^ h^−1^ under control and 574 ± 53 mg kg^−1^ h^−1^ under elevated CO_2_ conditions (*x*^2^ = 1.083, df = 14, *P* = 0.298) (Fig. 2B). Recovery time in bigfin reef squid was very similar between treatments, lasting an average of 8 hours and 8 minutes (±47 minutes) under control and 7 hours and 43 minutes (±1 hour and 13 minutes) under elevated CO_2_ conditions (*x*^2^ = 0.144, df = 14, *P* = 0.704) (Fig. 3B). The individual with the lowest aerobic scope (342 mg kg^−1^ h^−1^) also performed the fewest number of jets during the exercise protocol (11 jets). It is possible this individual, from the control treatment, did not achieve a true *Ṁ*O_2Max_ resulting in the lowest aerobic scope. The recovery time of bigfin reef squid was significantly longer than that of pygmy squid by an average of 7 hours (*x*^2^ = 113.080, df = 31, *P* < 0.001). A power analysis revealed a power of 0.146 on the comparisons of aerobic scope in bigfin reef squid between CO_2_ treatments.

## Discussion

After extended exposure to elevated CO_2_ levels, we found no significant changes to any measures of oxygen consumption in either the two-toned pygmy squid (*I. pygmaeus*) or the bigfin reef squid (*S. lessoniana*) when compared with current-day control CO_2_ conditions. The responses observed here under elevated CO_2_ differ from the reduction in *Ṁ*O_2Max_ and *Ṁ*O_2Routine_ observed in the jumbo squid (Rosa and Seibel, 2008) and the decrease in oxygen uptake rates observed in the common cuttlefish and European squid during late-stage embryo incubation (Rosa *et al*., 2014; Sigwart *et al.*, 2016). However, these results are consistent with those of juvenile common cuttlefish (Gutowska *et al.*, 2008) and experiments by Hu *et al*. (2014) in which bigfin reef squid showed no changes in *Ṁ*O_2Routine_ under CO_2_ levels of 1585 μatm after a 7 day exposure period. We predicted that elevated CO_2_ would interfere with oxygen extraction causing a decrease in the *Ṁ*O_2Max_, reducing aerobic scope. However, the recent estimation that CO_2_ levels of ~ 1000 μatm would cause a drop in squid hemocyanin-O_2_ saturation by no more than 1.6% (Birk *et al*., 2018) suggests that squid may be able to cope with elevated CO_2_ without costs to their oxygen uptake capabilities. Our results indicate that the respiratory physiology of the two species of tropical cephalopod studied here are likely to be resilient to realistic future CO_2_ levels in the habitats they currently inhabit.

Based on our results and from studies previously conducted on other cephalopod species, it appears that elevated CO_2_ elicits a range of aerobic responses in cephalopods, which may be life stage dependent. The reduced oxygen uptake observed during the embryonic period in some cephalopods at elevated CO_2_ (Rosa *et al*., 2014; Sigwart *et al.*, 2016) might be expected, as acid–base regulatory mechanisms in cephalopods often remain rudimentary until respiration switches from cutaneous (via skin) to branchial (via gills) (Hu *et al*., 2011b). Although little is known about the acid–base regulatory capabilities of pygmy squid, those of bigfin reef squid have been thoroughly investigated. Bigfin reef squid, among other cephalopods, have evolved ion regulatory epithelia in both the gills (Hu *et al.*, 2011a, 2014) and skin cells (Hu *et al.*, 2011b, 2013), which are effective in coping with acid-base disturbances.

Acid–base regulatory abilities are an indispensable trait in cephalopods, as well as in all animals, as there is a continuous natural confrontation with respiratory CO_2_ that can cause extra- and intra-cellular pH disturbances (Robertson, 1949; Hu *et al.*, 2013). Some cephalopods have been shown to have advanced acid–base regulatory machinery, comparable to that of fishes, and can effectively regulate their acid–base balance at high levels of CO_2_ without compromising aerobic capacities (Gutowska *et al.*, 2008, 2010). It appears that this may also be the case for both pygmy squid and bigfin reef squid, as *Ṁ*O_2_ remains unchanged at CO_2_ levels (~1000 μatm) projected for the end of the century under the business as usual CO_2_ emissions scenario. There could be changes in the allocation of resources at elevated CO_2_, such as towards acid–base regulation, but this does not appear to affect the aerobic performance of squids. However, there may still be energetic costs at much higher CO_2_ levels, because the extreme CO_2_ treatment (4134 μatm) used by Hu *et al*. (2014) resulted in a 40% reduction in *Ṁ*O_2Routine_ in bigfin reef squid.

The recovery times of pygmy squid and bigfin reef squid observed under control CO_2_ conditions were noticeably different between species. While pygmy squid recovered in an average recovery time of less than 1 hour, bigfin reef squid took an average of ~8 hours to return to their *Ṁ*O_2Routine_. During trials, all pygmy squid were observed to use the adhesive gland on their mantle to remain attached to the wall of the respirometry chamber throughout the duration of the measurement period. In contrast, bigfin reef squid lack this gland and maintained a suspended position by the use of their undulating fins throughout the trials. This unique mechanism in pygmy squid grants the species a much greater capacity for rest than in the continuously swimming bigfin reef squid. This may help to explain why pygmy squid, under control conditions, overcame excess post-exercise oxygen consumption more rapidly than bigfin reef squid. Furthermore, the *Ṁ*O_2Routine_ values measured for pygmy squid are likely closer to true standard metabolic rates, i.e. maintenance costs, than those of bigfin reef squid due to their mode of life.

Most squid are negatively buoyant, and the requirements of being `at rest’ while remaining suspended in the water column still have considerable aerobic costs (Bartol *et al.*, 2001). As bigfin reef squid recover from exercise, they remain relatively active in order to remain suspended in the water column. The species has large fins that run the full length of the mantle that can be used to maintain neutral buoyancy without the use of jetting. This allows them to spend, perhaps, a smaller fraction of their energy budget to maintain neutral buoyancy when compared to other squid species with proportionally much smaller fins, requiring fin use to be coupled with jetting (Hu *et al.* 2014). While this decoupled option of swimming with fin undulations alone in bigfin reef squid is more efficient than in more powerfully swimming pelagic squid species, pygmy squid can attach to a piece of seagrass or other benthic structure where they may truly rest, thus avoiding spending extra energy during recovery. This mode of rest could also be very beneficial to pygmy squid because lower mantle ventilation pressures in squid, such as those during rest, as opposed to those during active swimming, lead to higher oxygen extraction rates (Melzner *et al*. 2006).

It is worth noting that the average values of *Ṁ*O_2Routine_ values in bigfin reef squid under control CO_2_ levels observed by Hu *et al*. (2014) were markedly higher, by ~70%, than those observed here. The *Ṁ*O_2Routine_ values in this experiment were determined during a 22-hour trial period, whereas the previous experiment determined *Ṁ*O_2Routine_ during a 20–30 minute measurement period. The results from this experiment indicate that bigfin reef squid experience excess post-exercise oxygen consumption for ~8 hours after exhaustive exercise. While Hu *et al*. (2014) did not exercise the squid in their experiments, it is likely that a measurement period of 20–30 minutes may not have allowed for a true *Ṁ*O_2Routine_ measurement due to the handling stress and introduction to the respirometry chamber (Keys, 1930; Svendsen *et al*., 2018). Nevertheless, considering all animals were handled and introduced to the chambers in the same way, the comparisons among CO_2_ treatments from the previous experiment are still useful. Furthermore, there were comparable results upon exposure to similar elevated CO_2_ levels, causing unaltered *Ṁ*O_2_ in both the former experiments and in those that were performed here.

In this study, we used a relatively prolonged exposure to elevated CO_2_ (>20% of the animals lifespan) along with intermittent-flow respirometry to determine the effects of elevated CO_2_ on cephalopod *Ṁ*O_2_. In juvenile jumbo squid, *D. gigas*, elevated CO_2_ levels (estimated at ~ 1000 μatm) suppressed *Ṁ*O_2Max_ by ~ 30% and *Ṁ*O_2Routine_ by ~ 20% (Rosa and Seibel, 2008). However, with a longer acclimation to CO_2_ and the use of intermittent-flow respirometry rather than flow-through respirometry, there was no effect of elevated CO_2_ (1410 μatm) on this species (Birk *et al*., 2018). Furthermore, Hu *et al*. (2014) found different effects of elevated CO_2_ on *S. lessoniana* depending on the length of exposure. This suggests that future studies should use exposure times that are long enough for individuals to overcome any short-term effects of elevated CO_2_ as well as consider intermittent-flow respirometry methods (e.g. Steffensen, 1989). Future studies should also consider additional environmental changes, such as elevated temperature, which could potentially interact with elevated CO_2_ to affect oxygen consumption in unexpected ways. Multifactorial experiments will be important to gain a more complete understanding of the effects of climate change and ocean acidification on cephalopod physiology. Furthermore, power analyses revealed that the sample size of this study was low considering the variation among individuals. A larger sample size would be needed to investigate more subtle effects of elevated CO_2_ that may not have been detected in this study.

Our findings contribute to increasing evidence suggesting a level of tolerance to elevated CO_2_ in adult cephalopods, both among species and among the traits being tested. It has been suggested that the active, high-energetic lifestyle, along with occurrences of natural hypercapnia during the course of embryonic development, constitutes factors that pre-adapt cephalopods to cope with elevated CO_2_ levels (Melzner *et al.*, 2009b). While elevated CO_2_ has been shown to illicit negative physiological responses in some cephalopod species (e.g. Kaplan *et al.*, 2013; Sigwart *et al.*, 2016), other studies have observed no adverse effects of elevated CO_2_ on the same traits in other species (e.g. Gutowska *et al.*, 2010; Birk *et al*., 2018). Physiological stress from elevated CO_2_ during the early ontogeny of animals has been described as the `true bottleneck’ through which species must pass in order to successfully tolerate future elevated CO_2_ oceans, even for species that demonstrate tolerance in other traits (Melzner *et al.*, 2009b). Therefore, it will be important for future studies to investigate the effects of elevated CO_2_ on cephalopods during their embryonic and paralarval development, especially in species that show no adverse effects of elevated CO_2_ in other physiological traits as adults, such as those studied here.

## Conclusions

This study shows that *Ṁ*O_2Max_, *Ṁ*O_2Routine_, aerobic scope and recovery time of two tropical cephalopod species are unaltered following prolonged exposure to elevated CO_2_. In warmer tropical latitudes, where the energy requirements of cephalopods are higher, altered aerobic performance could be particularly consequential. However, it appears that CO_2_ levels projected for the end of this century will not adversely affect the respiratory performance of either the two-toned pygmy squid or bigfin reef squid. The unaltered aerobic performance of these ecologically distinct cephalopod species, from separate taxonomic orders, after prolonged exposure to elevated CO_2_ indicates that a wide range of cephalopod species may have the aerobic capacity to cope with an increasingly CO_2_-rich ocean. However, as some previous studies have observed negative effects of elevated CO_2_ on respiratory performance of some cephalopod species, it will be important to understand the potentially species-specific and life stage-specific effects, which can have critical implications for the structure of marine ecosystems in the future.

## Supplementary Material

Ch3_script_coz024

Figure_S1_coz024
