# Supplementary material for: Aerobic performance of two tropical cephalopod species unaltered by prolonged exposure to projected future carbon dioxide levels
Source: Conserv Physiol. 2019 Jun 7;7(1):coz024. doi: 10.1093/conphys/coz024 (PMC6554595; doi:10.1093/conphys/coz024)
Supplement: Ch3_script_coz024 [file ch3_script_coz024.docx]

**###Pygmy Squid**

**##Max O2 uptake**

#boxplot

ggplot(PY, aes(y=MAX, x=Treat))+

geom_boxplot()+

geom_point(colour='red')

#Shapiro-Wilk normality test for data

shapiro.test(PY$MAX)

#model

m1=lmer(MAX~Treat+JETS+INKS+(1|Tank), data=PY)

#Shapiro-Wilk normality test for residuals

shapiro.test(resid(m1))

#plot1

plot(m1)

#plot2

autoplot(m1, which=1:6)

#Summary analysis

summary(m1)

#ANOVA

Anova(m1)

**##Routine O2 uptake**

#boxplot

ggplot(PY, aes(y=REST, x=Treat))+

geom_boxplot()+

geom_point(colour='red')

#Shapiro-Wilk normality test for data

shapiro.test(PY$REST)

#model

r1=lmer(REST~Treat+JETS+INKS+(1|Tank),data=PY)

#Shapiro-Wilk normality test for residuals

shapiro.test(resid(r1))

#plot1

plot(r1)

#plot2

autoplot(r1, which=1:6)

#Summary analysis

summary(r1)

#ANOVA

Anova(r1)

**##Aerobic scope**

#boxplot

ggplot(PY, aes(y=Scope, x=Treat))+

geom_boxplot()+

geom_point(colour='red')

#Shapiro-Wilk normality test for data

shapiro.test(PY$Scope)

#model

s1=lmer(Scope~Treat+JETS+INKS+(1|Tank),data=PY)

#Shapiro-Wilk normality test for residuals

shapiro.test(resid(s1))

#plot1

plot(s1)

#plot2

autoplot(s1, which=1:6)

#Summary analysis

summary(s1)

#ANOVA

Anova(s1)

**##Recovery time**

#boxplot

ggplot(PY, aes(y=EPOCH, x=Treat))+

geom_boxplot()+

geom_point(colour='red')

#Shapiro-Wilk normality test for data

shapiro.test(PY$EPOCH)

#model

e1=lmer(EPOCH~Treat+JETS+INKS+(1|Tank),data=PY)

#Shapiro-Wilk normality test for residuals

shapiro.test(resid(e1))

#plot1

plot(e1)

#plot2

autoplot(e1, which=1:6)

#Summary analysis

summary(e1)

#ANOVA

Anova(e1)

**##Power analysis for aerobic scope**

#Cohen’s D

M1=746

M2=775

S1=146

S2=154

Cohen.d= (M1 - M2)/sqrt(((S1^2) +(S2^2))/2)

pwr.t2n.test(

n1=8,

n2=10,

d=Cohen.d,

sig.level=0.05,

power=NULL

)

**###Bigfin Reef Squid**

**##Max O2 uptake**

#boxplot

ggplot(BF, aes(y=MAX, x=Treatment))+

geom_boxplot()+

geom_point(colour='red')

#Shapiro-Wilk normality test for data

shapiro.test(BF$MAX)

#model

m1<-glm(log(MAX)~Treatment+JETS+INKS,data=BF,family=gaussian(link="identity"))

#Shapiro-Wilk normality test for residuals

shapiro.test(resid(m1))

#plot1

plot(m1)

#plot2

autoplot(m1, which=1:6)

#Summary analysis

summary(m1)

#ANOVA

Anova(m1)

**##Routine O2 uptake**

#boxplot

ggplot(BF, aes(y=RESTING, x=Treatment))+

geom_boxplot()+

geom_point(colour='red')

#Shapiro-Wilk normality test for data

shapiro.test(BF$RESTING)

#model

r1=glm(log(RESTING)~Treatment+JETS+INKS,data=BF,family=’gaussian’)

#Shapiro-Wilk normality test for residuals

shapiro.test(resid(r1))

#plot1

plot(r1)

#plot2

autoplot(r1, which=1:6)

#Summary analysis

summary(r1)

#ANOVA

Anova(r1)

**#Aerobic scope**

#boxplot

ggplot(BF, aes(y=SCOPE, x=Treatment))+

geom_boxplot()+

geom_point(colour='red')

#Shapiro-Wilk normality test for data

shapiro.test(BF$SCOPE)

#model

S1<-glm(log(SCOPE)~Treatment+JETS+INKS,data=BF,family=gaussian(link="identity"))

#Shapiro-Wilk normality test for residuals

shapiro.test(resid(s1))

#plot1

plot(s1)

#plot2

autoplot(s1, which=1:6)

#Summary analysis

summary(s1)

#ANOVA

Anova(s1)

**#Recovery time**

#boxplot

ggplot(BF, aes(y=EPOC, x=Treatment))+

geom_boxplot()+

geom_point(colour='red')

#Shapiro-Wilk normality test for data

shapiro.test(BF$EPOC)

#model

e1=glm(log(EPOC)~Treatment+JETS+INKS,data=BF,family=gaussian(link="identity"))

#Shapiro-Wilk normality test for residuals

shapiro.test(resid(e1))

#plot1

plot(e1)

#plot2

autoplot(e1, which=1:6)

#Summary analysis

summary(e1)

#ANOVA

Anova(e1)

**##Power analysis for aerobic scope**

#Cohen’s D

M1=508.66

M2=574.05

S1=122.05

S2=139.03

Cohen.d= (M1 - M2)/sqrt(((S1^2) +(S2^2))/2)

pwr.t2n.test(

n1=9,

n2=7,

d=Cohen.d,

sig.level=0.05,

power=NULL

)
