# Supplementary material for: Aerobic performance of two tropical cephalopod species unaltered by prolonged exposure to projected future carbon dioxide levels
Source: Conserv Physiol. 2019 Jun 7;7(1):coz024. doi: 10.1093/conphys/coz024 (PMC6554595; doi:10.1093/conphys/coz024)
Supplement: Figure_S1_coz024 [file figure_s1_coz024.docx]

Figure S1 – An example from preliminary results of *Ṁ*O_2_ over time for *I. pygmaeus*. This individual recovered from *Ṁ*O_2Max_ to *Ṁ*O_2Routine_ in approximately 2 hours.
